# “Self” and “other”: A conceptual bridge linking normal with pathological personality

**DOI:** 10.3389/fpsyt.2022.1023236

**Published:** 2022-10-20

**Authors:** Richard C. Howard

**Affiliations:** Institute of Mental Health, University of Nottingham, Nottingham, United Kingdom

**Keywords:** self, personality, psychopathology, narrative identity, two polarities model, motivation

## Abstract

The goal of this paper is to try and close the gap between the ways in which pathological and normal personality, including their development, are conceptualized. To this end, attention is drawn to parallels that exist between the ways self-function is conceptualized in contemporary personality psychology and in recent iterations of the major psychiatric nosologies, particularly ICD-11. Conceptualizations in both normal and abnormal personality see a fundamental dichotomy between *self as identity* and *self as socially interdependent* (vs autonomous). Evidence is reviewed supporting a basic dichotomy between two categories of personality pathology that can be subsumed under the labels “Acting Out” and “Anxious-Inhibited.” It is suggested that fundamental to the personality pathology subsumed under “Acting Out” is a deficient interdependent self, while a defective self-identity is proposed to underlie the personality pathology subsumed under “Anxious-Inhibited.”

## Introduction

In this paper an attempt is made to draw together thinking about the self seen in contemporary personality theory and in recent iterations of the major psychiatric nosologies. We first outline contemporary theories of normal personality and its development that have emphasized different aspects of self, in particular a duality of self: self as *identity* and self as *interpersonal*. We then briefly review recent advances in the conceptualization of abnormal personality (“personality disorder”). We show that a disordered self represents a central focus of current attempts to define personality pathology. In the following section of the paper we propose a distinction drawn by Blackburn and colleagues (1) between two empirically derived types or categories of personality pathology, “Acting Out” and “Anxious/Inhibited.” Under these two categories can be subsumed most if not all the traditional PD categories listed in DSM-5. It is argued that “Acting Out” and “Anxious/Inhibited” can be interpreted as reflecting disturbances in the two main types of self identified in normal personality: interpersonal and identity, respectively. The goal of this paper is to close the gap that separates the ways in which personality pathology and normal personality are conceptualized. It is argued that self as identity (self as autobiographical narrator) and self as socially interdependent can be seen as themes that are common to both abnormal and normal personality.

### Self and relatedness: Two superordinate dimensions of normal personality

Contemporary theories of personality have delineated different aspects of self. For example, in their two-polarities model of personality, Fan et al. (2) argue for two major structural elements of personality, self (identity), and relatedness. Self and relatedness are the carriers that achieve the dual developmental functions of independence and interdependence. They are mirrored in two fundamental developmental dimensions: *interpersonal relatedness*—the *development of increasingly mature, intimate, mutually satisfying, and reciprocal interpersonal relationships*—and *self-definition*—*the development of an increasingly differentiated, integrated, realistic, and essentially positive sense of self or identity* [(2), p. 3]. Personality is said by the authors of this model to have two basic functions in relation to the internal and external environments. The first is *to maintain independence*, achieve ego functional autonomy, and then construct self-identity. This independence or autonomy helps a person meet their needs for achievement and power. The second function is *to connect a person with their social environment* by assuming social roles such as father, brother, friend, colleague, or leader and to then *meet their needs for affiliation and intimacy*.

Other authors have drawn a similar distinction between an independent or autonomous self and an interdependent self. Baumeister (3), for example, asserts that an interdependent self is firmly embedded in a network of social relationships complete with obligations and accomodations. In contrast, the independent self is an autonomous self-contained agent operating on its own, making choices and pursuing self-selected goals complete with an inner set of values and preferences. Both Fan et al. and Baumeister emphasize the relative salience of self and relatedness in Western and Eastern cultures, respectively. Fan et al. (2) comment: *Western cultures emphasize the inherent separateness of distinct people, who must be independent from others and realize and express their unique attributes*…… *Eastern cultures, which can be represented by China, emphasize the fundamental connectedness between human beings* (p. 7).

Fan et al. draw a further distinction between *intra*personal and *inter*personal relatedness. The former reflects *how individuals think about their social world*—their social cognition; the latter defines how a person relates to the social world through behavior or performance. The distinction here is between an individual’s internal thoughts and feelings about their social world, in contrast to how they manifest to others in their interpersonal behavior.

### Self in DSM-5 and ICD-11

Recent iterations of the Diagnostic and Statistical Manual (DSM) and the International Classification of Diseases (ICD) have both emphasized self and other deficits as core aspects of personality dysfunction. Criterion A of the alternative (hybrid) model outlined in section 3 of DSM-5, requires for a PD diagnosis the presence of a moderate or greater impairment in personality functioning, defined by the degree to which there is an intact sense of self—a clear, coherent identity, and effective self-directedness—and interpersonal functioning—reflecting a good capacity for empathy and for mature, mutually rewarding intimacy with others (4). Likewise, ICD-11 defines four features of personality disorder that define its severity along a dimension ranging from mild to severe (see Table 1). Important to note is the emphasis that ICD-11 gives to self-dysfunction (criterion 1) and interpersonal dysfunction (criterion 2). Important too in criterion 1 is an implied motivational deficit: an inability to plan, choose and implement appropriate goals. We note that both ICD-11 and the DSM-5 alternative model, but particularly the former, emphasize the dimensionality of PD in terms of its severity as manifested in the degree of self and other dysfunction. Discussing the merits of the ICD-11 PD model, Clark et al. (5) suggest it represents a significant change in the conceptualization of personality pathology in two major ways: first, by changing from a set of discrete categories to a fully dimensional perspective; and second, by causing us to think of personality pathology as having the following two components:

**TABLE 1:** Aspects of personality functioning in ICD-11 that contribute to severity determination in Personality Disorder (25).

**(1) Degree and pervasiveness of disturbances in functioning of aspects of the self** ○ Stability and coherence of one’s sense of identity (e.g., extent to which identity or sense of self is variable and inconsistent or overly rigid and fixed). ○ Ability to maintain an overall positive and stable sense of self-worth. ○ Accuracy of one’s view of one’s characteristics, strengths, limitations. ○ Capacity for self-direction (ability to plan, choose, and implement appropriate goals). **(2) Degree and pervasiveness of interpersonal dysfunction across various contexts and relationships (e.g., romantic relationships, school/work, parent-child, family, friendships, peer contexts).** ○ Interest in engaging in relationships with others. ○ Ability to understand and appreciate others’ perspectives. ○ Ability to develop and maintain close and mutually satisfying relationships. ○ Ability to manage conflict in relationships. **(3) Pervasiveness, severity, and chronicity of emotional, cognitive, and behavioral manifestations of the personality dysfunction** ○ Tendency to be emotionally over- or under-reactive, and having difficulty recognizing unwanted emotions (e.g., does not acknowledge experiencing anger or sadness) ○ Distortions in the accuracy of situational and interpersonal appraisals under stress (e.g., dissociative states, psychotic-like beliefs or perceptions, and paranoid reactions). ○ Behavioral responses to intense emotions and stressful circumstances (e.g., propensity to self-harm or violence). **(4) The extent to which the dysfunctions in the above areas are associated with distress or impairment in personal, family, social, educational, occupational or other important areas of functioning.**

1. impairment in personality *functioning*—one might say in one’s personhood itself —that is, a general failure to mature adaptively and to develop the capacity to live successfully in one’s world.
2. the more specific ways in which personality impairment is manifest, that is, an individual’s basic maladaptive-range personality traits.

One should note the close similarity between this description of personality functioning, in particular “a general failure to mature adaptively,” and the definition of interpersonal relatedness in Fan et al.’s two polarities model of personality: the *development of increasingly mature, intimate, mutually satisfying, and reciprocal interpersonal relationships.*

### Relatedness and identity: Two superordinate dimensions of abnormal personality

As a result of detailed analysis of PD symptoms in mentally disordered offenders, Blackburn et al. (1) were able to identify two high-order factors, Acting Out and Anxious-Inhibited. PD was assessed using the International Personality Disorder Examination (IPDE). Symptoms contributing significantly to each factor, together with their loadings, can be seen in Table 1 in Howard et al. (6). “Acting Out” combines features of antisocial PD (including conduct disorder), narcissistic PD (particularly grandiose), and histrionic PD together with externalizing symptoms of borderline PD (anger and impulsivity). Anxious-Inhibited combines elements of avoidant, borderline (internalizing symptoms) and dependent PDs together with neurotic (vulnerable) forms of narcissism.

**FIGURE 1:**
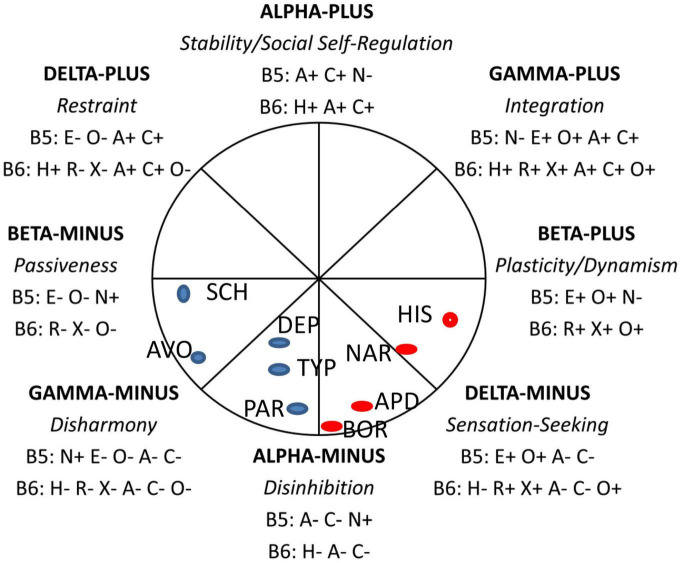
The circumplex of personality metatraits. B5, Big Five traits: N, Neuroticism/Emotional Stability; E, Extraversion; O, Openness to Experience/Intellect; A, Agreeableness; C, Conscientiousness. B6, Big Six traits: H, Honesty-Humility/Propriety; R, Resiliency/Emotionality; X, Extraversion; A, Agreeableness; C, Conscientiousness; O, Originality/Openness to Experience. +, positive pole of the trait; –, negative pole of the trait. The approximate positions of PDs, as reported by Zawadzki (8), are indicated by blue and red circles: SCH, Schizoid PD; AVO, Avoidant PD; DEP, Dependent PD; TYP, Schizotypal PD; PAR, Paranoid PD; BOR, Borderline PD; APD, Antisocial PD; NAR, Narcissistic PD; HIS, Histrionic PD. The figure is based on Strus and Cieciuch (26) and reprinted with permission.

The division between PDs subsumed under the “Acting Out” vs. “Anxious-Inhibited” dichotomy is supported by an analysis of PDs carried out within the framework of the circumplex model of personality metatraits [CPM: (7)], a circumplex constituted by two orthogonal dimensions: Alpha/Stability and Beta/Plasticity (see Figure 1). CPM integrates the Big Five of the Five Factor Model of personality and the HEXACO model, allowing the integration of models of temperament, emotion, motivation, values, wellbeing, and mental health problems, including personality disorders, into a single framework. Results of a meta-analysis carried out by Zawadzki (8) showed that all PDs subsumable under the “Acting Out” umbrella—histrionic, antisocial, narcissistic, and borderline—clustered together within the lower right quadrant of the circumplex shown in Figure 1 (beta plus, delta minus, and alpha minus). In contrast, all the PDs that would be subsumed under the “Anxious-Inhibited” umbrella, namely schizoid, avoidant, dependent, schizotypal, and paranoid, fell within the lower-left quadrant shown in Figure 1, defined by beta minus, gamma minus, alpha minus. Common to this lower-left quadrant is high Big Five Neuroticism (B5N+ in Figure 1) and (in general) low Big Five Extraversion (B5E− in Figure 1).

Several points are worthy of note here. First, borderline PD is situated at the interface between the two quadrants, consistent with it straddling the divide between “Acting Out” and “Anxious-Inhibited.” Second, alpha minus, which the authors identify with *social antagonism*, is common to both sets of personality disorders, consistent with high hostility being a core feature of both “Acting Out” and “Anxious-Inhibited.” Social antagonism is said by these authors to capture externalizing, e.g., anger, aggression, and internalizing, e.g., spitefulness, vindictiveness, or envy problems, together with antisocial tendencies and disregard for social norms and other people. Third, Delta minus is identified with sensation seeking and low impulse control, while gamma minus is associated with low self-motivation, “a competence that is the basis for the capacity to strengthen motives related to the attainment of broadly defined goals and intentions, for example, values, personal standards, or commitments” (9). This lack of self-motivation echoes the low self-direction component of the self-dysfunction domain in ICD-11. Importantly, this distinction implies distinct self-regulation deficits associated with “Acting Out” and “Anxious-Inhibited.” In the case, of “Acting Out,” the deficit consists in a lack of impulse control associated with risk-taking, reward/sensation-seeking, and what Clark et al. (5) refer to as “reckless impulsivity.” In the case of “Anxious-Inhibited,” the self-regulation deficit, consisting in a lack of self-direction, is most likely linked to emptiness/anhedonia and associated amotivation (10). Last, we should note that the “Anxious-Inhibited” type of personality pathology is characterized, as its name implies, by social inhibition. The key location for social inhibition within the CPM (see Figure 1) is the pole Beta Minus/Passiveness which encompasses submissiveness and dependency in interpersonal relations, but also self-consciousness which seems to be crucial for controlling and inhibiting behavior in social situations (11). These authors define social inhibition as a psychosocial disposition that has five components: (i) social interactions that are related to evaluation and/or are unfamiliar (situational context); (ii) contingent and generally lowered self-esteem, which is dependent on social reinforcements. Consequently, it manifests in (iii) a preoccupation with being evaluated by others and monitoring one’s own behavior; (iv) feeling uncertainty and tension, and (v) avoiding social exposure, attention, and evaluation through limiting goal-directed activity or taking self-protective behavior (behavioral component).

At the core of both “Acting Out” and “Anxious-Inhibited” is high hostility/antagonism, manifested, in the case of “Acting Out,” by a hostile/dominant interpersonal style, in the case of “Anxious Inhibited,” by a hostile/submissive interpersonal style (1). At the core of “Acting Out” is an interpersonal deficit characterized by an unwillingness to interact with others in an empathic way [“prosocial apathy”: (12)]. People who score high on “Acting Out” are motivated by interpersonal malevolence, e.g., a desire to control others (desire for power) or to hurt, harm or exploit them. They are motivated to avoid empathy by downregulating feelings of concern for others (13). In terms of the above-mentioned distinction between autonomous and interdependent selves, autonomy predominates over interdependence, helping these individuals to meet their malevolent goals (power over, and exploitation of, others), at the expense of being disconnected from their social environment.

### Narrative identity

At the core of “Anxious-Inhibited” are self deficits that include, in particular, a disturbed narrative identity. This refers to the autobiographical story that we construct about ourselves [“self as autobiographical author”: (14)], linking together in a coherent fashion our past, present, and future. Narrative identity is often the route by which subjective self-continuity is established and maintained (15). A review of recent literature on narrative identity in PD acknowledges that current knowledge about narrative identity and PD is based primarily on community samples of predominantly females with BPD (16). With this caveat, the authors state that, taken together, narrative identity research paints a compelling picture of the *subjective sense of self in (B)PD as fragmented, defective, non-agentic, confused, and emotionally isolated*. Lind (15) particularly emphasizes the presence in borderline PD of deficient autobiographical reasoning, a reflective process in which the story is organized and evaluated to create a temporally, causally, and thematically coherent account of the person’s life. Lind suggests that autobiographical reasoning may be particularly disturbed in individuals with severe PD, whose stories may be severely disorganized and culturally detached, with a lack of causal connections, and thematic connections that are either absent or encompass themes of severe thwarted agency and communion.

## Discussion

The goal of this paper is to try and close the gap between the ways in which personality pathology and normal personality (and its development) are conceptualized. To this end we have drawn attention to parallels that exist between self-dysfunction as conceptualized in recent iterations of the major psychiatric nosologies, particularly ICD-11, and conceptualizations of self in contemporary personality psychology. Conceptualizations in both normal and abnormal personality see a fundamental dichotomy between self as identity and self as socially interdependent (vs. autonomous). We have reviewed evidence for a basic dichotomy between two categories of personality pathology that, following Blackburn et al., can be subsumed under the labels “Acting Out” and “Anxious-Inhibited.” We suggest that fundamental to the personality pathology subsumed under “Acting Out” is a deficient interdependent self, while a defective self-identity underlies the personality pathology subsumed under “Anxious-Inhibited.”

It is important to note that these categories, “Acting Out” and “Anxious-Inhibited,” are not viewed as disjunctive (either…. or) but rather as conjunctive. That is, one could score high on either, neither, or both. Since scores on “Anxious-Inhibited” and “Acting Out” have been found separately to correlate with PD severity (6), the most severe personality pathology would be expected in individuals who score high on both dimensions, for example those in whom features of both antisocial PD and borderline PD are combined (17).

We noted above the presence of maladaptive-range personality traits in ICD-11. These comprise the five trait domains of negative affectivity, disinhibition, dissociality, anankastia, and detachment. Here we should note three aspects of personality traits that tend to be overlooked. The first is that they require a particular type of situation to trigger their expression in overt behavior. This has been acknowledged by scholars of prosocial behavior, who have advocated an affordance-based framework that considers situational features as providing opportunities for the expression of certain aspects of personality in behavior (18). Despite the suggestion of Hepp and Niedtfeld (19) that research in PDs might benefit from adopting an affordance-based framework, the importance of situational factors has been relatively neglected by PD researchers. One exception is a recent study that looked at the situations encountered by individuals (university students) in relation to DSM-5 alternative model personality traits (20). Results showed substantive relations between personality pathology and situational experiences, and these associations were overwhelmingly driven by subjective situational *construal* as opposed to situation contact. While promising, this study requires replication in a clinical or forensic sample. In short, unique situational experiences appear to be differentially driven by different aspects of personality pathology.

The second important, and often overlooked, aspect of personality traits is their link to motivation and goal-directed behavior. Within mainstream personality psychology there is an increasing integration of motivation and personality (21). Some authors acknowledge that traits are inseparable from goals. McCabe and Fleeson (22), for example, review findings supporting an explanation of traits in terms of their utility: they permit the individual to focus on the pursuit and achievement of a certain set of goals. An example might be high antagonism, where possessing the trait arguably allows the individual to pursue goals of hedonic self-gratification, even (or especially) when this is at the expense of other people. A lack of fear (or high boldness) in combination with antagonism would be particularly conducive to the achievement of self-serving hedonic goals such as excitement or sexual gratification. Although ICD-11 does at least acknowledge lack of goal direction as one aspect of the self-deficit in PD, a similar integration of motivation and personality needs to occur in the field of personality pathology. One way in which this integration might occur is highlighted by Lind (15). This author points out that motivational and affective themes, reflecting what the narrator is striving for and how these experiences are evaluated emotionally, are commonly found in the autobiographical narratives of people with PD, particularly borderline patients. Lind cogently argues that narrative identity is a crucial aspect that remains to be incorporated within dimensional approaches to PD.

Finally, we should mention the objections expressed by some authors to the view that maladaptive traits are central to personality pathology. For Sharp and Vanwoerden (23), for example, coherence and consistency of the personal narrative is what determines healthy or unhealthy personality function—not the presence or absence of maladaptive traits *per se*. Thus underlying maladaptive personality traits is a dysfunctional self. This contention receives support from recent work on empathy by Krol and Bartz (24). These authors showed that a clear, coherent, and stable sense of self is important for empathic responding, particularly when, in its mature form, it is characterized by low empathic distress together with high empathic concern. One question that remains largely unaddressed is how a synergy evolves developmentally between maladaptive traits and self-dysfunction.

We conclude that “self” and “other” themes have shown a parallel development in theoretical accounts of both normal and abnormal personality. The approach advocated here sees personality pathology as dichotomized into two overarching types, “Anxious-Inhibited” and “Acting Out,” characterized by “self” and “other” deficits respectively.

## Author contributions

The author confirms being the sole contributor of this work and has approved it for publication.
